# PaDef (*Persea americana* var. drymifolia), a Plant Antimicrobial Peptide, Triggers Apoptosis, and Induces Global Epigenetic Modifications on Histone 3 in an Acute Lymphoid Leukemia Cell Line

**DOI:** 10.3389/fmolb.2022.801816

**Published:** 2022-01-24

**Authors:** Paola Jiménez-Alcántar, Rodolfo López-Gómez, Joel E. López-Meza, Alejandra Ochoa-Zarzosa

**Affiliations:** ^1^ Centro Multidisciplinario de Estudios en Biotecnología, Facultad de Medicina Veterinaria y Zootecnia, Universidad Michoacana de San Nicolás de Hidalgo, Morelia, Mexico; ^2^ Instituto de Investigaciones Químico-Biológicas, Universidad Michoacana de San Nicolás de Hidalgo, Morelia, Mexico

**Keywords:** plant defensin, apoptosis, Leukemia, PaDef defensin, epigenetic modifications, Histone deacetylase inhibitor

## Abstract

In recent years, it has been recognized that epigenetic alterations play an important role in the development and maintenance of cancer, including leukemias. Furthermore, it is known that these alterations are involved in the emergence of resistance to conventional chemotherapeutics. Consequently, molecules with an anticancer activity whose activity is ruled by epigenetic modifications are attractive to search for new therapies against cancer. The plant antimicrobial peptides have been widely evaluated as molecules with anticancer activity; however, the analysis of the epigenetic regulation induced by these molecules associated with this activity is scarce and still is an unexplored field. In this work, we show that the PaDef defensin, a plant antimicrobial peptide from Mexican avocado fruit (*Persea americana* var. drymifolia) is cytotoxic for Jurkat cell line from acute lymphoid leukemia cells, through an apoptotic process. PaDef inhibited cell viability in a concentration-dependent manner, with an IC_50_ = 47.3 μM. Treatment of Jurkat cells with PaDef (IC_50_) induced cell death by apoptosis dependent on caspases 8 and 9; besides, it was related to an increase in the production of reactive oxygen species and the loss of mitochondrial membrane potential. Interestingly, the inhibition of caspase activation by inhibitors of caspases 8 and 9 does not revert the reduction in viability, suggesting that other mechanisms, in addition to caspase activity, could be participating in the PaDef cytotoxic effect. Also, the modifications in the histone 3 tails induced by PaDef in Jurkat cells were evaluated, specifically acetylation and methylation. PaDef increased global histone 3 acetylation and lysine 9 specific marks (2-fold and up to 4-fold, respectively). These effects correlated with the reduction of the Histone Deacetylase activity (HDAC, ∼50%). Based on methylation marks, PaDef treatment increased lysine 9 di- and tri-methylation tags (2-fold in both cases). The epigenetic modulation induced by PaDef on Jurkat cells could be related to the chromatin compaction-decompaction promoting gene expression or repression; however, further studies are necessary to correlate these marks with the transcription of specific genes. Therefore, the study of new molecules that may have anticancer activity through epigenetic modulation is interesting.

## Introduction

Cancer remains a serious threat worldwide. According to GLOBOCAN (2020), leukemias represent the 15th number of diagnosed cancer and the 11th place in cancer deaths worldwide. Data of the International Agency for Research on Cancer (IARC) of the World Health Organization (WHO) reports in 2018 around 430,000 new cases of leukemia worldwide and 300,000 deaths for this cancer (International Agency for Research on Cancer, 2020). Of the total of leukemia cases, 75% correspond to cases of Acute Lymphoblastic Leukemia (ALL) (International Agency for Research on Cancer, 2020).

ALL arises from progressive malignant transformation and uncontrolled proliferation of lymphoid hematopoietic progenitor cells, which accumulate in the bone marrow, blood, and extramedullary sites (Terwilliger and Abdul-Hay, 2017). The two main groups of this pathology are B-cell ALL and T-cell ALL, and 80% of the total cases occur in children (Terwilliger and Abdul-Hay, 2017). The majority of cases (85–90%) correspond to B-cell ALL, which generally has a good prognosis and a high cure rate (Bhojwani et al., 2015); however, when relapses are reported (10–15%) the disease usually culminates in early mortality (Wu and Li, 2018). The remaining percentage of total ALL cases (10–15%) corresponds to T-cell ALL that have a poor prognosis, with high relapse rates and low chances of recovery (Bhojwani et al., 2015). On the other hand, ALL in young and older adults has a worse prognosis with increasing age. The prognosis in older adults is poor because comorbidities prevent the administration of established chemotherapy regimens. Also, alterations that generate resistance to chemotherapeutic treatments can often occur (Aldoss et al., 2019). Furthermore, relapse in ALL is still a major cause of death related to this cancer for all ages (Roberts, 2018). In recent years, conventional chemotherapy has increased cure rates in ALL pediatric patients by up to 90% in developed countries (Paul et al., 2016). However, no substantial progress has been made to improve the cure rate in adults. Besides, the side effects of chemotherapy (myelosuppression, hepatotoxicity, cardiotoxicity) reduce the quality of life of patients and/or may put them at risk. Likewise, highlights the fact that conventional chemotherapy is still insufficient to treat the entire population that presents the disease, and the search for new therapeutic strategies is essential (Wu and Li, 2018).

During the last few years, therapies with an epigenetic approach have emerged as a promise to improve responses to conventional chemotherapeutic treatments. It has been recognized that epigenetic alterations not only play a fundamental role in the initiation and development of cancer but also are fundamental for the appearance of chemoresistance. Unlike genetic mutations, epigenetic alterations are reversible, this fact makes them an attractive target for the search for epigenetic therapies against cancer (Gambacorta et al., 2019; Hajji et al., 2021).

Antimicrobial peptides (AMPs) are attractive molecules as an alternative for cancer treatment, due they are cytotoxic for tumor cells, show lower development of resistance, and have certain specificity towards cancer cells (Deslouches and Di, 2017). AMPs are produced by a great diversity of organisms (animals, plants, bacteria, fungi, protists, and archaea) as an essential component of their innate immune response (Sinha and Shukla, 2019). AMPs usually are short cationic molecules, ranging from 10 to 50 aa long and structurally form α-helices, β-sheets, extended structures, and loops (Deslouches and Di, 2017; Haney et al., 2017). The main function associated with AMPs is to protect against pathogens; however, immunomodulatory functions (Hazlett and Wu, 2011), mitogenic (Wong et al., 2006), antiangiogenic (Van Zoggel et al., 2012), and antitumor properties (Ren et al., 2012; Guzmán-Rodríguez et al., 2016; Flores-Alvarez et al., 2018) have been described. To date, 3257 AMPs have been reported (https://aps.unmc.edu/), of which about 7.7% have a cytotoxic activity to cancer cells. Noteworthy, some AMPs are not toxic to normal mammalian cells and have a broad spectrum of cytotoxic activity on tumor cells. Also, it has been described the epigenetic modulation of AMPs related to anticancer activities, as is the case of lunasin, a peptide derived from soy that inhibits the acetylation of Histone 3 and 4 (Wan et al., 2017; Janssens et al., 2019). This fact increases the relevance of the study of AMP as an attractive source of new therapies against cancer.

Plant defensins are cationic AMPs with 45–55 aa residues, notwithstanding their diversity in the aa sequence, these AMPs possess clear amino acid conservation in some of their positions, such as 8 cysteine residues that allow them to form 4 disulfide bridges. Plant defensins are expressed in seeds, leaves, roots, barks, pods, tubers, fruits, and flower organs and their main functions involve antifungal and antibacterial activities; however, some defensins have a toxic effect on cancer cells without harming healthy cells (Guzmán-Rodríguez et al., 2015; Parisi et al., 2018). Previously, we have shown that defensin γ-Thionin from *Capsicum chinense*, a plant antimicrobial peptide with cytotoxic activity towards MCF-7 breast cancer cells, modulates global Histone 3 acetylation and methylation marks, increasing the lysine 9 acetylation mark (H3K9Ac) and lysine 9 dimethylation mark (H3K9me2) (Arceo-Martínez et al., 2018). However, the role of epigenetic modulation of AMPs in their cytotoxic activity is still an understudied field.

In previous work, we demonstrated that the PaDef defensin from Mexican avocado (*Persea americana* var. drymifolia) is cytotoxic to MCF-7 breast cancer cells, through the induction of apoptosis intrinsic pathway (Guzmán-Rodríguez et al., 2016). On the other hand, the cytotoxic effect of the peptide was tested in the hematological cell line K562 from chronic myeloid leukemia, inducing apoptosis through the extrinsic pathway (Flores-Alvarez et al., 2018). Additionally, the peptide has been tested in peripheral blood mononuclear cells without showing a cytotoxic effect (Guzmán-Rodríguez et al., 2016; Flores-Alvarez et al., 2018).

In the present work, we show that PaDef is cytotoxic to the acute lymphoid leukemia cell line Jurkat, activating the extrinsic and intrinsic pathways of apoptosis. Furthermore, we assessed whether the PaDef cytotoxicity was related to epigenetic modifications in the histone 3 (H3), specifically the global and lysine 9 (K9) acetylation marks, as well as changes in the di- and trimethylation (me2 and me3) marks of H3 lysine 9.

## Materials and Methods

### Peptide

The peptide used in this work corresponds to the mature region of PaDef (ATCETPSKHFNGLCIRSSNCASVCHGEHFTDGRCQGVRRRCMCLKPC, 47 aa) (Genbank KC007441) (Guzmán-Rodríguez et al., 2013). This peptide was chemically synthesized and obtained from BIOMATIK. The peptide at a concentration of 962 μM was resuspended in 20% dimethyl sulfoxide (DMSO), and then air oxidized for 5 days at room temperature for disulfide bond formation. For all of the experiments, the final concentration of vehicle (DMSO) was 0.98%, which was also used as a control. The peptide was used in a concentration range of 9.6–58 μM in agreement with the results observed from a previous study in the leukemia cell line K562 (Flores-Alvarez et al., 2018). Primary and secondary structures of the peptide are shown in Supplementary Figure S1.

### Jurkat Cell Culture

The Human Jurkat cell line was obtained from American Type Culture Collection (ATCC). Cells were cultured in RPMI-1640 medium (Sigma) supplemented with 12.5% fetal bovine serum (v/v) (FBS, Corning) and 100 U/mL penicillin and streptomycin (Gibco), in an atmosphere of 5% CO_2_ and at 37°C.

### MTT Viability Assay and Determination of the Half-Maximal Inhibitory Concentration (IC_50_)

Jurkat cells (2 × 10^4^ cells/well) were cultured in 96-well plates and synchronized in RPMI-1640 medium by serum deprivation for approximately 18 h. Cells were then treated with PaDef peptide 9.6–58 μM and DMSO (0.98%, control) and incubated for 24 h as reported (Flores-Alvarez et al., 2018). After incubation with the treatments, 10 μL of MTT solution (5 mg/mL, Sigma) in phosphate-buffered saline (PBS 138 mM NaCl, 2.7 mM KCl, 10 mM Na_2_HPO_4,_ and 10 mM KH_2_PO_4_) was added to each well and incubated for 4 h at 37°C. Finally, formazan crystals were dissolved with acidic isopropanol (100 μL, 95% isopropanol, and 5% 1N HCl). Absorbance measurements were performed in a microplate reader (Bio Rad) at 595 nm. Actinomycin D (0.5 µM, Act-D) (Cayman Chemical) was used as a positive control for cell death. The half-maximal inhibitory concentration (IC_50_) was calculated by linear regression analysis using the program GraphPad Prism 8. To corroborate the IC_50_ calculated, a flow cytometric assay was performed using SYTO 9 fluorescent green and propidium iodide for nucleic acid staining (Invitrogen). For the assay, 8 × 10^4^ cells/well were cultured in 24-well plates and synchronized with serum-free RPMI-1640 medium for 18 h and then treated for 24 h with the IC_50_ calculated and with vehicle (DMSO 0.98%). Finally, cells were stained with the fluorochromes according to the manufacturer’s instructions and analyzed in a BD Accuri™ C6 cytometer (BD Biosciences). For the rest of the experiments, the IC_50_ corroborated in this assay was used.

### Measurement of the Transmembrane Potential

Depolarization of the cellular transmembrane potential was assessed using the membrane potential sensitive dye 3,30-dipropylthiadiarbocyanine iodide, DiSC3 (5) (Sigma). For this, 2 × 10^4^ cells/well were cultured in 96-well black-bottom plates. Before the culture, cells were washed twice with Hanks HEPES buffer and resuspended with 100 µL of Hanks HEPES buffer containing DiSC3 (5) (200 μM), seeded into the wells of the plate, and incubated for 30 min in a CO_2_ incubator. PaDef 47.3 μM and DMSO were added to the assays in sextuplicate, and subsequent changes in fluorescence intensity were monitored for 2 h on a Varioskan spectrophotometer (Thermo Scientific). Valinomycin was used as a positive control (Sigma, 200 μM).

### Calcium Efflux Testing

Calcium flux was assessed in a BD Accuri ™ C6 flow cytometer (BD Biosciences) using the calcium assay kit (BD Biosciences) according to the manufacturer’s instructions. For the assay, 1 × 10^5^ cells were used for each treatment. Cells were synchronized for 18 h in RPMI-1640 medium without serum and then incubated with the indicator dye for 1 h. The flow cytometry readings were set with a baseline fluorescence of 1 min, then PaDef 47.3 μM and control (DMSO 0.98%) treatments were added, the fluorescence intensity was monitored for another 4 min. 4alpha-Phorbol 12-myristate 13-acetate (3 mM; PMA, Sigma) was used as a positive control.

### Caspase Activity Assay

Assays were performed with 8 × 10^4^ cells/well in 24-well plates. Cells were synchronized for 18 h in RPMI-1640 medium without serum and then were treated for 24 h with PaDef 47.3 μM or vehicle (DMSO 0.98%). After this, cells were collected and stained according to the manufacturer’s instructions (kit CaspGLOW™ Fluorescein Active Caspase-8 or Caspase-9 Staining Kit). Caspase activity was measured through specific pharmacological inhibitors LEHD-FMK (inhibitor of caspase 9) and IETD-FMK (inhibitor of caspase 8) conjugated with fluorescein (FITC) that bind to the active enzyme. Actinomycin D (0.5 µM) was used as a positive control for cell death. The data were analyzed in a BD Accuri ™ C6 flow cytometer (BD Biosciences). Data were analyzed using the Accuri ™ C6 software.

### Assessment of Apoptosis

Assays were performed with 8 × 10^4^ cells/well in 24-well plates. Cells were synchronized and treated as described for caspase activity assays. After 24 h of treatment, cells were collected and stained according to the manufacturer’s instructions (annexin V, Alexa Fluor 488 conjugate, Invitrogen). The rate of apoptosis was determined using annexin V and 7AAD in a BD Accuri ™ C6 flow cytometer (BD Biosciences). Data were analyzed using FlowJo v10.4 software (TreeStar, Inc.). Actinomycin D (0.5 μM) was used as a positive control for apoptosis.

### Assessment of Mitochondrial Membrane Potential (ΔΨm)

Assays were performed with 8 × 10^4^ cells/well in 24-well plates and cells were synchronized and treated as described above. Cells were collected and stained with JC-1 dye (BD Biosciences) for 15 min at 37°C in the dark according to the manufacturer’s instructions. The cells were washed twice with assay buffer, and the fluorescence was measured in a BD AccuriTM C6 flow cytometer (BD Biosciences) using FlowJo software (TreeStar, Inc.). The JC-1 dye allows the differentiation of healthy cells (red fluorescence) from those with mitochondrial damage (green fluorescence).

### Determination of Reactive Oxygen Species

This assay was performed by flow cytometry in a BD Accuri ™ C6 flow cytometer (BD Biosciences) using DHE (dihydroethidium 5 μM, Molecular Probes) or DHR (dihydrorhodamine-123, 15 μM, Molecular Probes). Assays were performed with 8 × 10^4^ cells/well in 24-well plates. Cells were synchronized for 18 h in RPMI-1640 medium without serum and then treated with PaDef 47.3 μM or vehicle (DMSO 0.98%) for 24 h. Cells were collected and washed with PBS. Cells were resuspended in PBS with DHR or DHE and incubated for 1 h and 30 min, respectively, at 37°C in the dark. Ethanol 12% was used as a positive control. A total of 10,000 events were analyzed.

### Histone Extraction and Western Blot Analysis

Jurkat cells (1 × 10^6^ cells) were synchronized for 18 h by serum deprivation, then treated with PaDef 47.3 μM and vehicle (DMSO 0.98%) for 24 h. Then, they were washed 2 times with cold PBS and proceeded to histone extraction by acid extraction (Salgado-Lora et al., 2020). For this, the cells were resuspended in 1.5 mL of H-lysis solution (0.25 M sucrose, 3 mM CaCl_2_, 1 mM Tris pH 8, and 0.5% NP-40) and shook. Subsequently, the cell pellet was washed with 1 mL of H-wash solution (300 mM NaCl, 5 mM MgCl_2_, 5 mM DTT and 0.5% NP-40). Histone extraction was performed with the addition of 200 μL of H-extraction solution (0.5 M HCl, 10% glycerol, and 0.1 M 2-mercaptoethylamine-HCl), incubated on ice for 30 min. Further, cells were centrifuged at 13,000 rpm for 10 min at 4°C, the supernatant was recovered and cold acetone was added 1:5 ratio. Histones were allowed to precipitate for 5 days under refrigeration and the precipitate was recovered by centrifugation (13,000 rpm for 10 min) and dissolved in 20 μL of sterile deionized water and stored at −80°C until use.

For western blot analysis, histone samples were separated by electrophoresis on a 15% SDS-PAGE gel, and then transferred to a PVDF membrane using a semi-dry transfer unit (Fisher Scientific). For this, the gel was equilibrated for 15 min in transfer solution III (0.3 M Tris pH 10.4) and the membrane (previously hydrated in methanol) in solution II (0.25 M Tris pH 10.4). The transfer sandwich was assembled and allowed to run at 15 V for 1 h. The membranes were then blocked with 5% nonfat dry milk powder dissolved in cold PBS and left at 4°C overnight. The membranes were washed three times with cold TBS, and then the primary antibody (1:1,000) was added and incubated at 4°C overnight. The following antibodies against specific histone modifications were used: global acetylation (H3K9, K14, K18, K23, K27) (Abcam ab47915), H3K9ac (Abcam, ab10812) for acetylation, H3K9me2 (Abcam Ab1220), and H3K9me3 (Abcam, ab8898) for di- and tri-methylation, respectively, and antibody for histone H3 (Abcam, ab1791) was used for loading control. Subsequently, the membranes were incubated for 2 h at 4°C with horseradish peroxidase-coupled anti-IgG secondary antibody (1:3,000) (Cell Signaling Technology). The membranes were washed three times and 100 μL of WesternSure ECL substrate was added. Finally, the membranes were placed between two plastic sheets and exposed to an X-ray plate. The plate was manually developed in the dark. The intensity of the signals was quantified by densitometry with ImageJ software. 3.5 mM sodium butyrate was used as a positive control for H3K9 acetylation induction and negative for H3K9me2/3 methylation induction. Data were normalized based on H3 and presented as the relative level of expression for the vehicle.

### HDACs Activity Assay

Jurkat cells (1 × 10^6^ cells) were synchronized for 18 h by serum deprivation, then treated with PaDef 47.3 μM and vehicle (DMSO 0.98%) for 6 h. Then, they were washed 2 times with cold PBS, and preparation of samples for the fluorometric assay was performed according to the manufacturer’s instructions [Histone Deacetylase (HDAC) Activity Assay Kit Fluorometric Kit ab156064, Abcam]. The changes in fluorescence intensity were monitored for 1 h in a Varioskan spectrophotometer (Thermo Scientific).

### Cytotoxicity of Doxorubicin and the Combination With PaDef

The cytotoxicity of doxorubicin or its combination with PaDef was assessed by MTT assays. Jurkat cells (2 × 10^4^ cells/well) were cultured in 96-well plates and synchronized in RPMI-1640 medium by serum deprivation for approximately 18 h. Cells were then treated with doxorubicin 1–10 μM and incubated for 24 h. For the drug combination, the same aforementioned concentrations of doxorubicin and PaDef (9.6–87 μM) were used, and incubated for 24 h. Doxorubicin and PaDef were added simultaneously for the combination study. They were subsequently treated and analyzed as described in the MTT viability assay section.

Combination index (CI) was used to evaluate the combination effect based on IC_50_ values obtained from individual doxorubicin or PaDef, and the combined treatment (Chou and Talalay, 1984). CI was calculated according to the following equation, CI = (D)com_1_/(D)_1_ + (D)com_2_/(D)_2_ in which (D)com_1_ (or (D)com_2_) is the IC_50_ value for treatments 1(or 2) in the combination, (D)_1_ (or (D)_2_) is the IC_50_ value from individual treatment. The resulting combination index (CI) was then used to determine the outcome of the drug combination effect as (I) additive effect (CI = 1), (II) synergism (CI < 1), or (III) antagonism (CI > 1).

### Statistical Analysis

The data were analyzed with PRISM 8.0.2 software by performing a one-way analysis of variance (one-way ANOVA) and using the post hoc Tukey test. The results are reported as the mean ± the standard errors (SE), and the significance level was set at *p* ≤ 0.05. On the other hand, for western blot analysis, the Student’s t-test was used with a significance level of *p* ≤ 0.05.

## Results

### The Antimicrobial Peptide PaDef is Cytotoxic to Jurkat Acute Lymphoid Leukemia Cells

**FIGURE 1:**
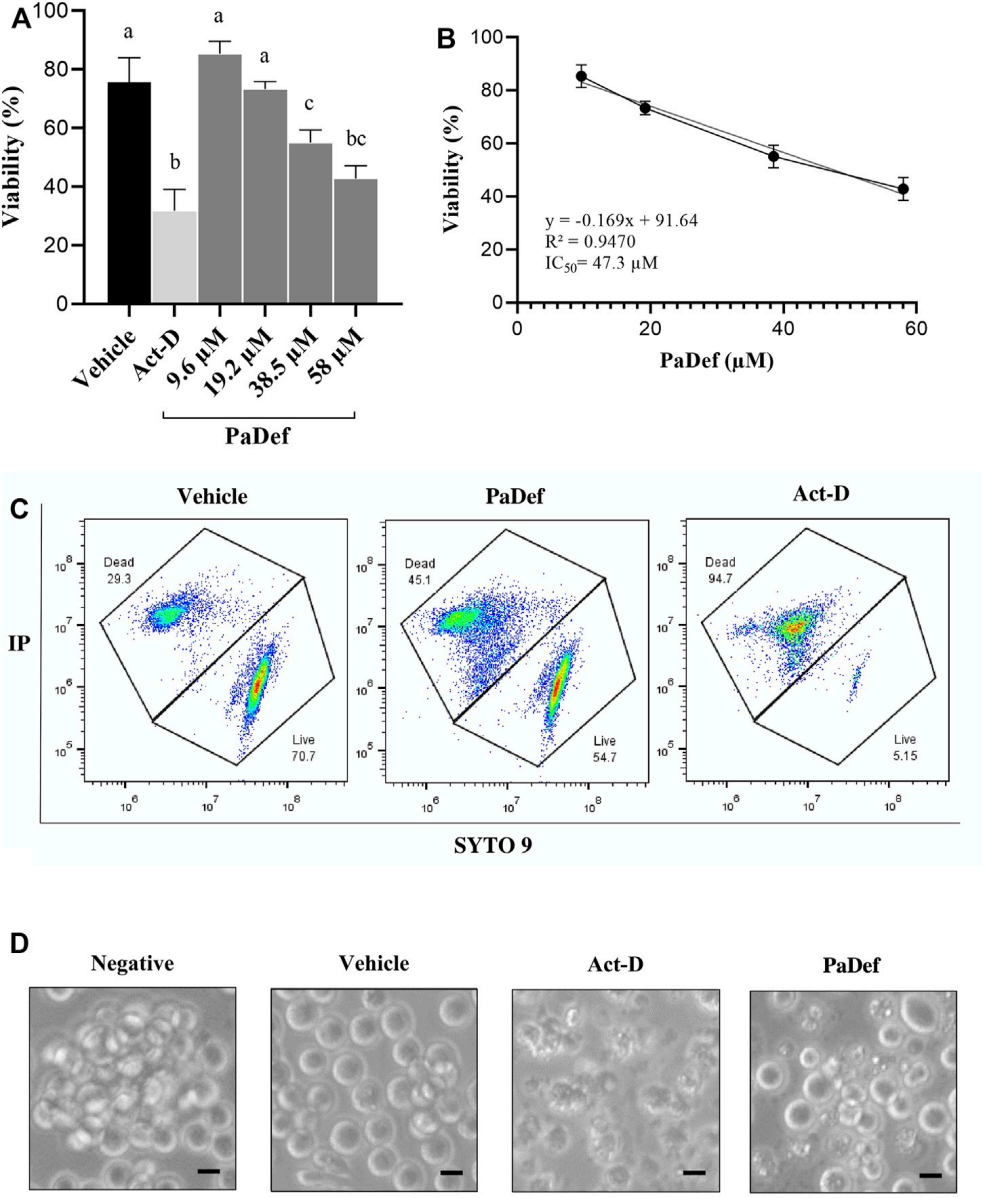
The antimicrobial peptide PaDef is cytotoxic to Jurkat acute lymphoid leukemia cells. **(A)** Effect of PaDef on the viability of Jurkat cells. Cells were treated under increasing concentrations of the peptide (9.6, 19.2, 38.5, and 58 μM) for 24 h and analyzed by MTT. The data show the percentage of cell viability. DMSO (0.98%) was used as a vehicle and Actinomycin D 0.5 μM (Act-D) as a positive death control. Data represent the mean of three independent experiments performed in triplicate. Different letters denote significant differences within the treatments (one-way ANOVA and Tukey´s comparison, *p* ≤ 0.05). **(B)** The half-maximal inhibitory concentration (IC_50_) was calculated by linear regression analysis; IC_50_: 47.3 μM; R^2^ = 0.9470. **(C)** Evaluation of PaDef IC_50_ by flow cytometry. Representative plots of the different treatment conditions are shown. Cells were treated with PaDef and vehicle (DMSO 0.98%), Actinomycin D 0.5 µM (Act-D) was used as a positive death control, and 10,000 events were analyzed. **(D)** Cell morphology of Jurkat after 24 h of treatment. Representative photographs taken by light-field microscopy are shown. Vehicle (DMSO 0.98%), PaDef 47.3 μM and Actinomycin D (Act-D) 0.5 μM. Scale bar 20 μm.

The effect of PaDef on the viability of the Jurkat cell line was analyzed by MTT assay. Increasing concentrations of the peptide (9.6, 19.2, 38.5 and 58 μM) were used for 24 h. Figure 1A shows that the cytotoxic effect of the peptide on the Jurkat cell line was in a concentration-dependent manner. From these results, the half-maximal inhibitory concentration (IC_50_) calculated was 47.3 μM (Figure 1B) and corroborated by flow cytometry (Figure 1C). For the rest of the experiments, this concentration was used. Also, cell morphology was analyzed by microscopy, evident alterations were observed in cells treated with PaDef and Actinomycin D (Figure 1D).

### The Antimicrobial Peptide PaDef Does Not Affect Membrane Integrity in Jurkat Cells

**FIGURE 2:**
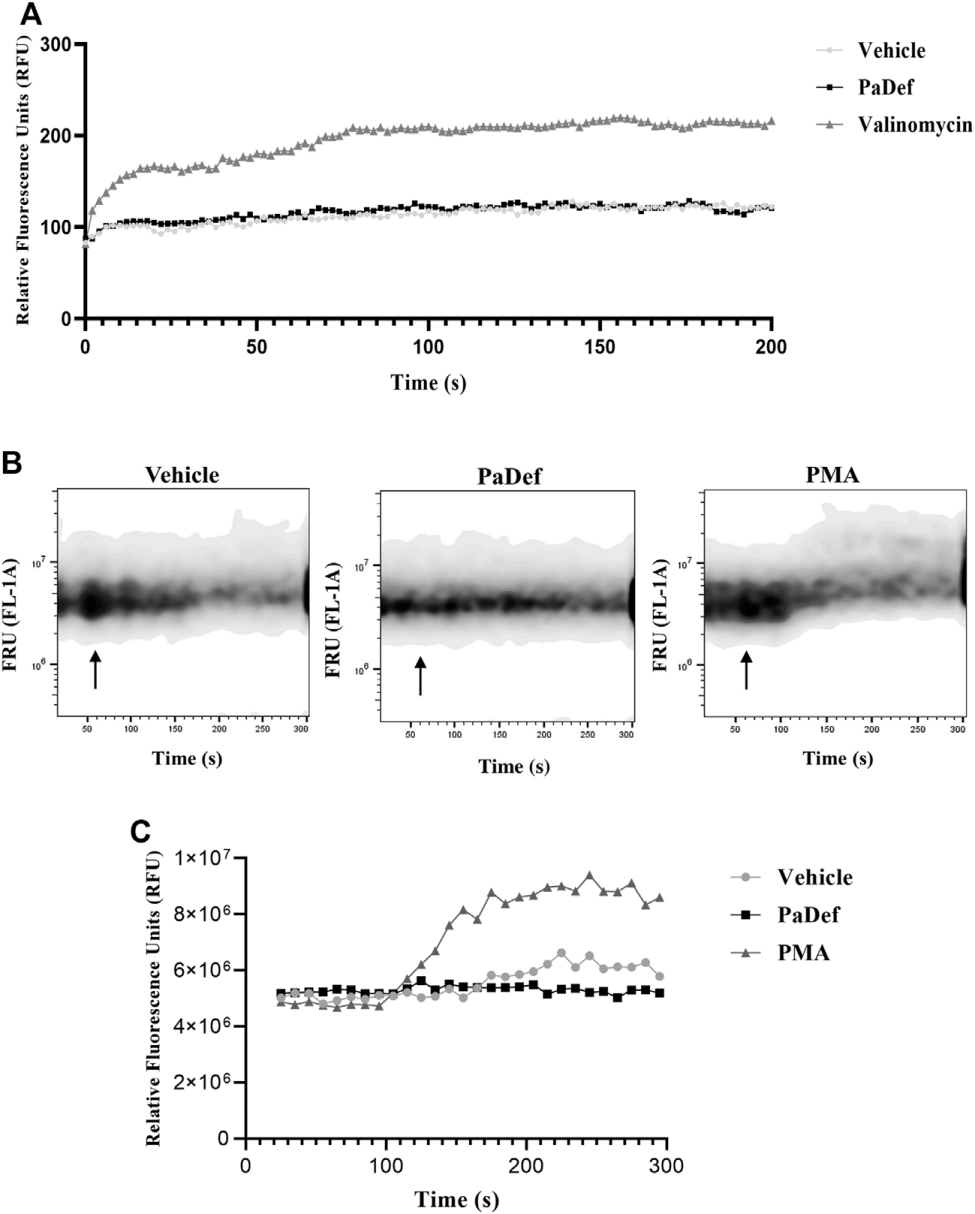
The antimicrobial peptide PaDef does not affect the membrane integrity of Jurkat cells. **(A)** Changes in membrane potential were analyzed with a dye sensitive to changes in membrane potential [DiSC3(5), 200 μM]. Cells were incubated with the dye for 30 min at 37°C, basal optical density was assessed and subsequently treatments: Vehicle (DMSO 0.98%), PaDef (47.3 μM), and Valinomycin (200 μM, positive control) were added and monitored for about 3.5 h with measurements every 2 min. **(B)** Cytosolic calcium efflux was analyzed by flow cytometry using the calcium assay kit (BD Biosciences). Measurements were performed for 5 min. For readings, a baseline fluorescence of 1 min was established, then the treatments were placed and the fluorescence intensity was monitored for another 4 min. The top panel shows the representative plots with the different treatments: Vehicle (DMSO 0.98%); PaDef (47.3 μM) and phorbol-myristate-acetate (c; PMA, Sigma) as a positive control. Arrows indicate the time at which the treatments were added. FRU (Fluorescence Relative Units). **(C)** The graph corresponds to the relative fluorescence units (RFU) of calcium release shown in (B).

One of the main described mechanisms of cytotoxic action of AMPs is the destabilization and disruption of the plasma membrane of cells. Therefore, the effect of PaDef on Jurkat cells membrane was evaluated. For this, membrane electrical potential and intracellular calcium efflux were assessed. The results indicated that PaDef treatment does not affect either the cell membrane potential (Figure 2A) or the calcium flux assay (Figures 2B,C). According to these results, the cytotoxic effect of PaDef on Jurkat cells does not involve damage to the plasma membrane.

### The Cytotoxic Effect of the Defensin PaDef on Jurkat Cells is Associated With Apoptosis Induction Through the Intrinsic and Extrinsic Pathways

**FIGURE 3:**
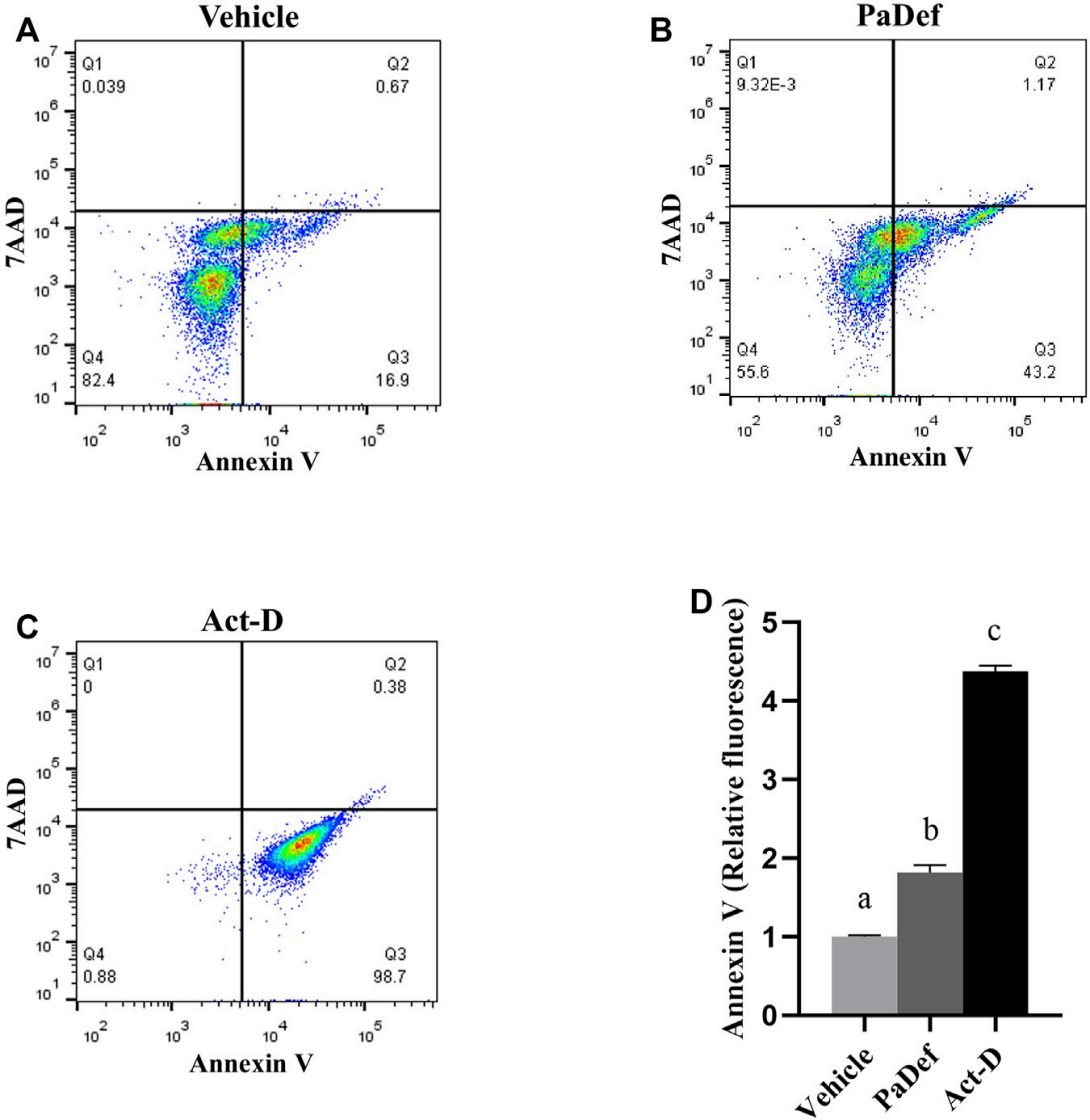
PaDef antimicrobial peptide induces apoptosis in Jurkat acute lymphoid leukemia line. Representative flow cytometry plots of the different treatments are shown; **(A)** Vehicle (DMSO 0.98%); **(B)** PaDef (47.3 μM); **(C)** Actinomycin D 0.5 μM (Act-D). Annexin V/7AAD staining was used to establish the apoptotic rate. Quadrants indicate viable cells (lower left quadrant), early apoptosis (lower right quadrant), late apoptosis (upper right quadrant), and necrotic cells (upper left quadrant). **(D)** The graph shows the relative fluorescence units for each condition. Each bar shows the mean of triplicates ± SE of three independent experiments. A minimum of 7,000 events per sample was collected. Different letters denote significant differences within the treatments (one-way ANOVA and Tukey´s comparison, *p* ≤ 0.05).

Plant AMPs have been described to induce death in cancer cells mainly through activation of apoptosis, but necrosis has also been reported (Guzmán-Rodríguez et al., 2015; Baxter et al., 2017). For this reason, we consider evaluating whether PaDef activates apoptosis in the Jurkat cell line. In Figure 3, representative plots of the flow cytometry assay with the treatments for 24 h are shown, in which it can be observed that PaDef induces apoptosis (upper and lower right quadrants of the plots) in the Jurkat cell line with a similar effect to Actinomycin D. In this same assay, necrosis (upper left quadrant of the plot) was evaluated using the 7AAD dye, the results indicated that PaDef does not induce this process in the cells.

**FIGURE 4:**
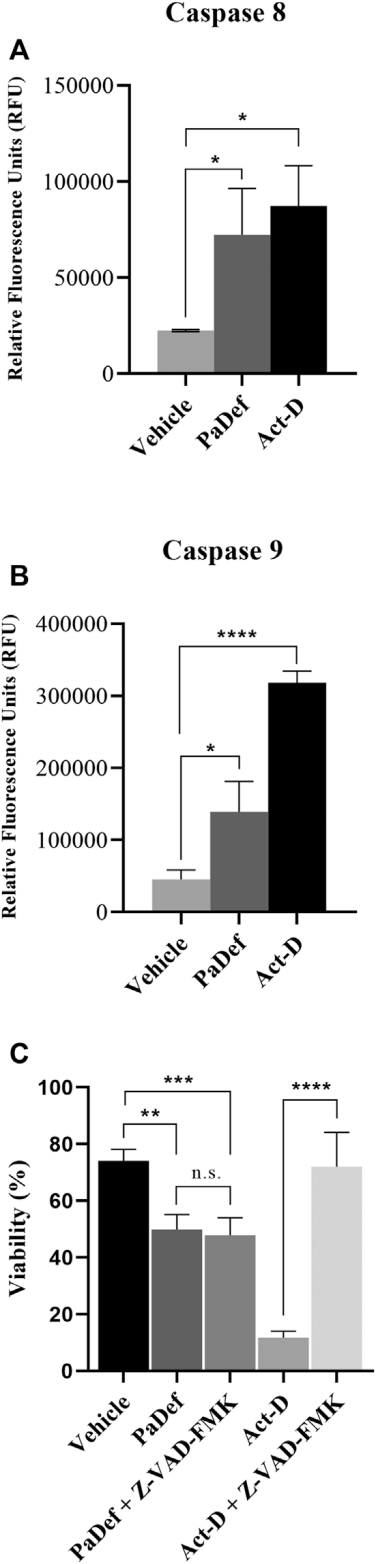
Antimicrobial PaDef peptide induces activation of caspases 8 and 9 in Jurkat cells. Cells were treated for 24 h with vehicle (DMSO 0.98%), PaDef (47.3 μM) and Actinomycin D (0.5 μM, Act-D). Cells were stained using FITC-IETD-FMK for caspase 8 **(A)** and FITC-LEHD-FMK for caspase 9 **(B)** and subsequently analyzed by flow cytometry. **(C)** Viability assay (MTT). Cells were treated for 24 h with vehicle (DMSO 0.98%), PaDef (47.3 μM), or Actinomycin D (0.5 μM) with or without Pan caspase inhibitor Z-VAD-FMK (added 1 h before the above treatments). Each bar shows the mean of triplicates ± SE of three independent experiments. * (*p* ≤ 0.05), ** (*p* ≤ 0.01), *** (*p* ≤ 0.001) and **** (*p* ≤ 0.0001) indicate the significant statistical difference compared between treatments (One-way ANOVA and Tukey’s pairwise comparison, *p* ≤ 0.05).

**FIGURE 5:**
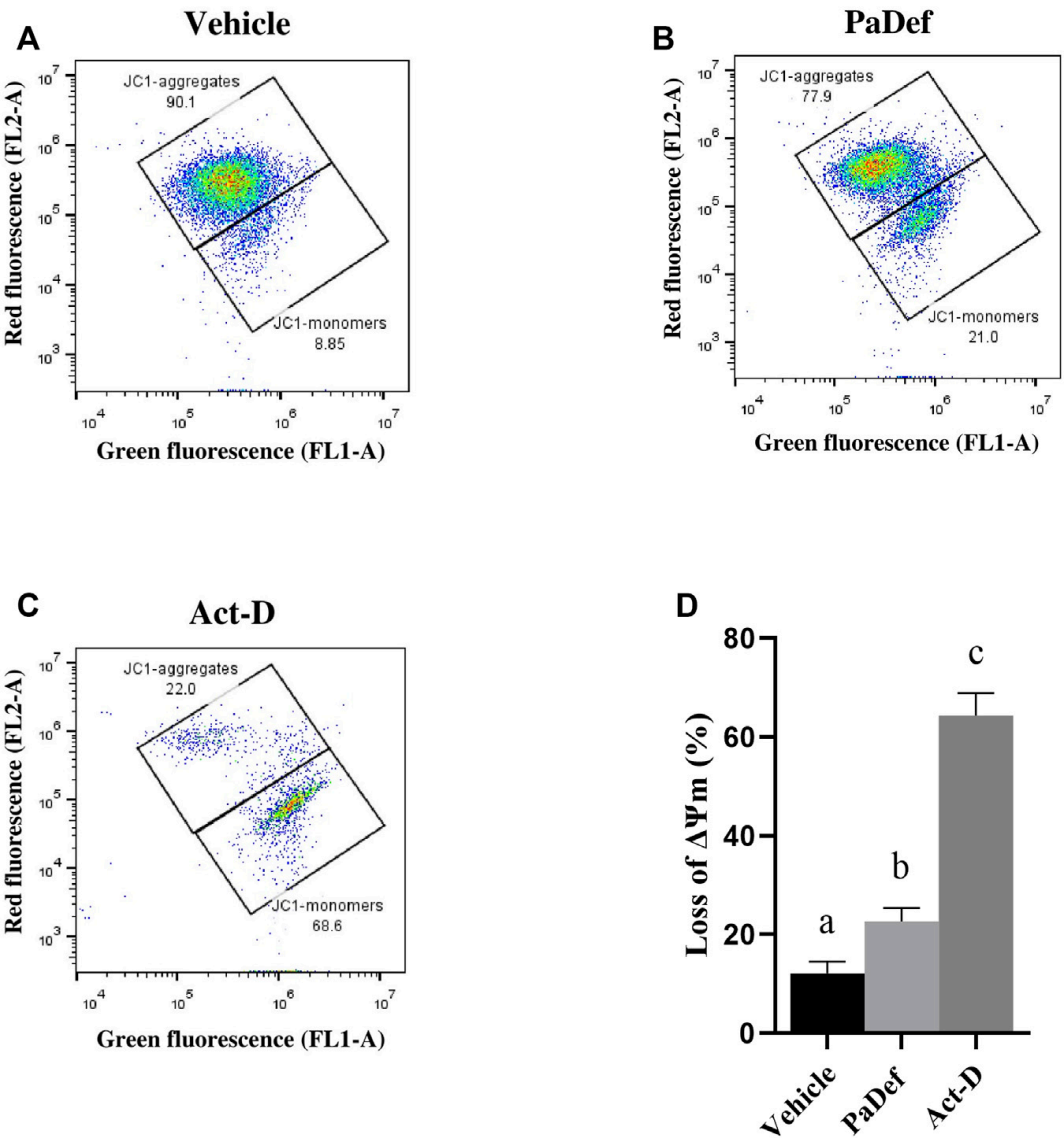
Antimicrobial PaDef peptide induces loss of mitochondrial membrane potential (ΔΨm) in Jurkat cells. Representative plots are shown. Cells were treated for 24 h with **(A)** vehicle (DMSO 0.98%), **(B)** PaDef (47.3 μM) or **(C)** Actinomycin D (0.5 μM, Act-D). Cells were stained with JC-1 dye and fluorescence was measured by flow cytometry. **(D)** The graph shows the percentage of loss of membrane potential for each treatment. Each bar shows the mean of triplicates ± SE of three independent experiments. Different letters denote significant differences (the same letter denotes no difference) in all values compared with each other (One-way ANOVA and Tukey’s pairwise comparison, *p* ≤ 0.05).

**FIGURE 6:**
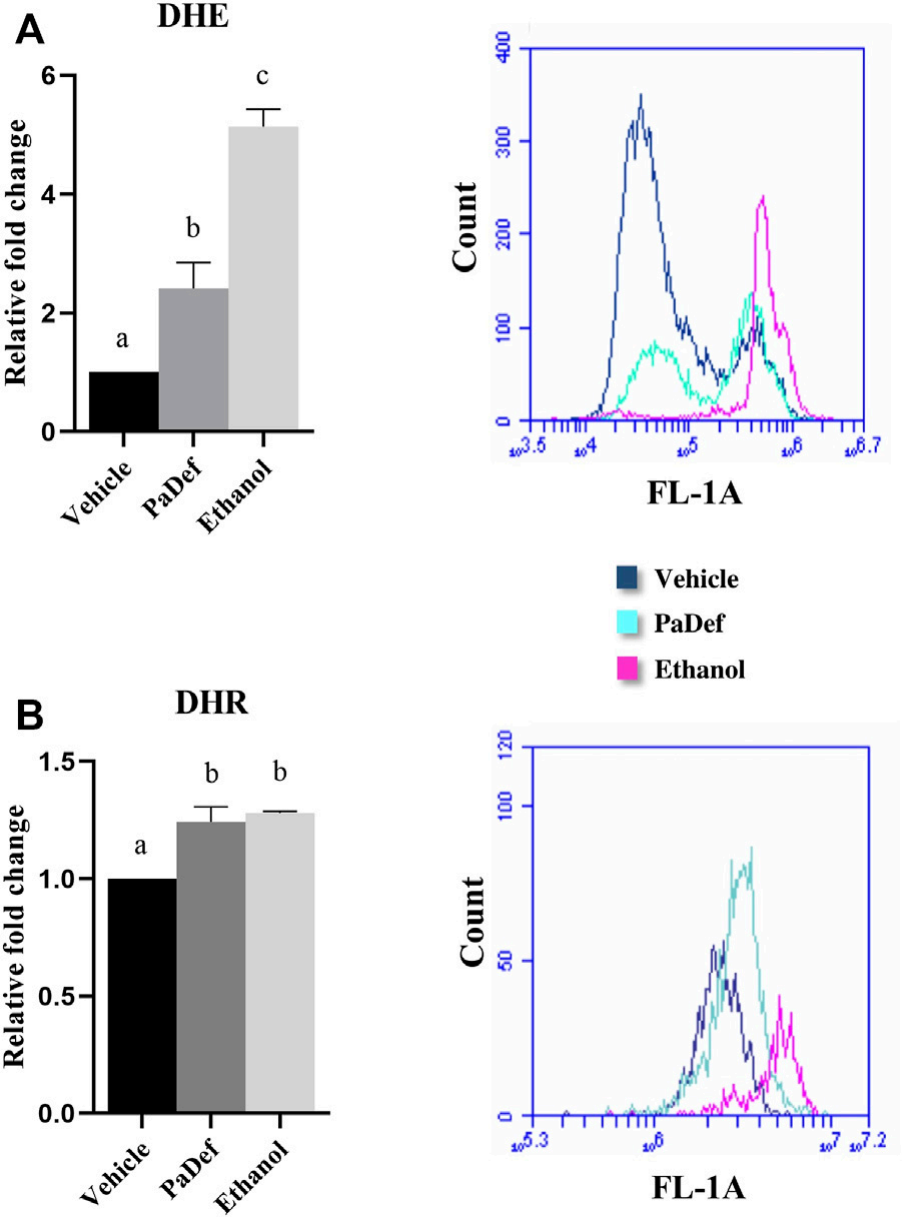
PaDef induces reactive oxygen species (ROS) generation in the Jurkat cell line. Cells were treated for 24 h: PaDef (47.3 μM), 0.98% DMSO Vehicle or 12% ethanol. Cells were subsequently stained with the indicators DHE (dihydroethidium) **(A)** and DHR (dihydrorhodamine) **(B)** and analyzed by flow cytometry. A representative image of a flow cytometry plot is shown. Each bar shows the mean of triplicates ± SE of two independent experiments. Different letters indicate statistically significant differences based on the vehicle (*p* ≤ 0.05) and were compared using one-way ANOVA and Tukey post hoc test (*p* ≤ 0.05).

Further, to determine the apoptosis pathway activated, the activity of caspases was assessed by flow cytometry. Jurkat cells treated with PaDef for 24 h showed activation of both caspase 8 and caspase 9 (Figures 4A,B). These results indicate that the peptide triggers apoptosis through both apoptosis pathways (intrinsic and extrinsic). Subsequently, by MTT it was determined if the inhibition of the activity of caspases blocks the death induced by PaDef. Jurkat cells treated 1 h with the Pan caspase inhibitor Z-VAD-FMK and then treated with PaDef (Figure 4C) showed a reduction in viability, which suggests that caspases are not the only mode of activation of apoptosis fired on these cells. Additionally, the activation of the intrinsic pathway of apoptosis was assessed by flow cytometry using the dye JC1, which is sensitive to changes in mitochondrial membrane potential. Cells treated with PaDef for 24 h showed a loss in mitochondrial membrane potential, suggesting that the intrinsic pathway of apoptosis is being activated (Figure 5). Also, it was evaluated if PaDef induces ROS production. The results indicated that PaDef induced the production of ROS, specifically the production of hydrogen peroxide (H_2_O_2_) (Figure 6A) and superoxide anion (O^2−^) (Figure 6B).

### The Antimicrobial Peptide PaDef induces Global Acetylation Modifications in Histone 3 by inhibiting HDACs

**FIGURE 7:**
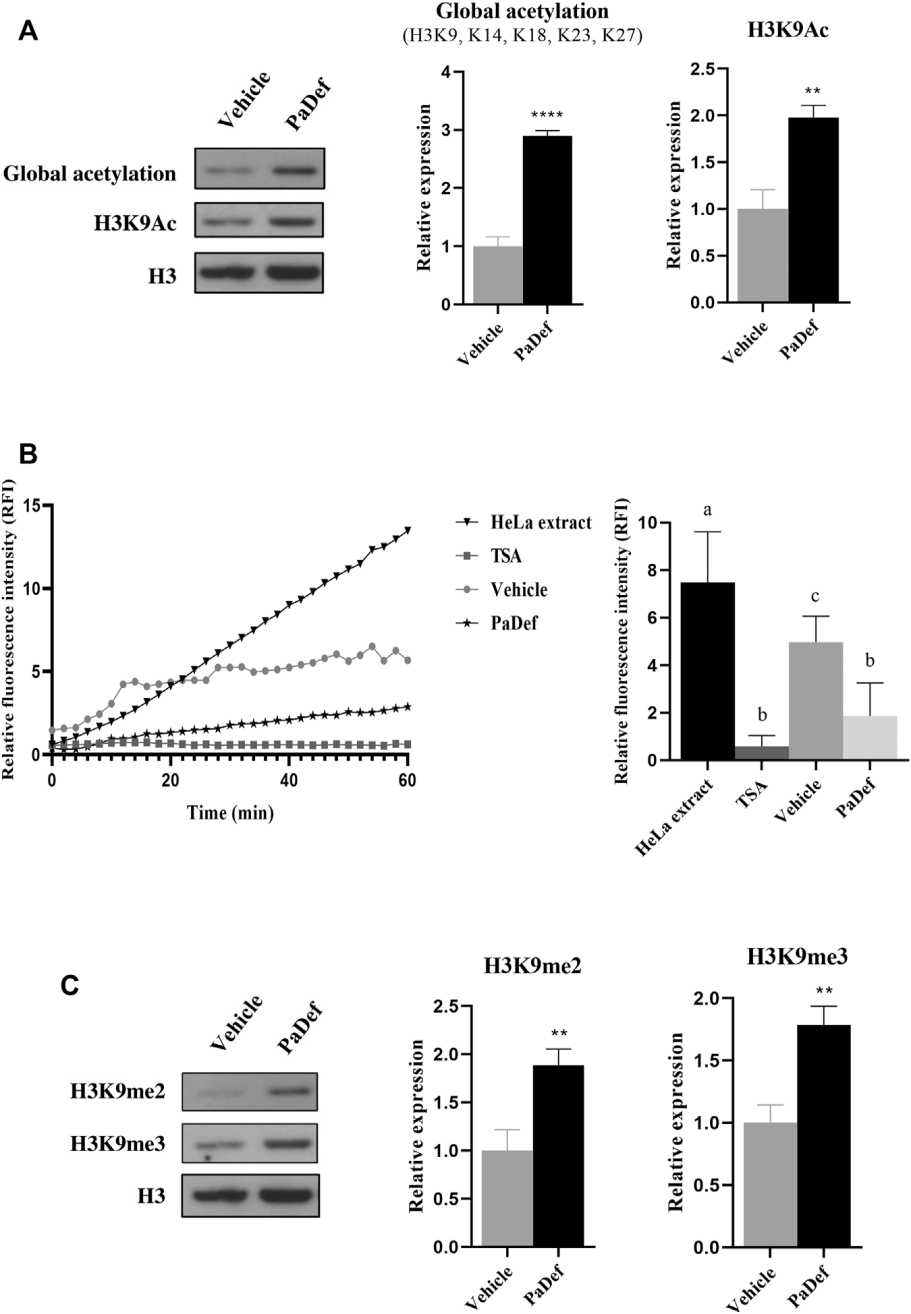
Epigenetic modifications induced by PaDef in Jurkat cells. PaDef induces global acetylation modifications in Histone 3 by inhibiting HDACs. **(A)** On the left side is a representative image of the western blot. Histones were extracted from cells treated for 24 h with PaDef (47.3 μM) or the corresponding vehicle (DMSO). Proteins were then separated by SDS-PAGE and western transfer assays were performed. Densitometric analysis plots corresponding to western blot analysis are shown on the right side. H3 levels were used as a loading control. Values were normalized to the vehicle (DMSO) for PaDef. ** (*p* ≤ 0.01) and **** (*p* ≤ 0.0001) indicate the significant statistical difference based on vehicle Student’s t-test. *n* = 4. Values represent the mean ± SE of four independent experiments. Vehicle DMSO (0.98%). **(B)** HDACs activity was measured with Histone Deacetylase (HDAC) Activity Assay Kit (Fluorometric) from Abcam ab156064. After 6 h of treatment with the peptide, nuclear protein extraction was performed and according to the manufacturer’s instructions, basal fluorescence was measured, and, subsequently, measurements were made every 2 minutes for 1 h. Each point shows the mean ± SE of triplicates of three independent experiments. A HeLa nuclear extract (Abcam kit) was used as a positive control for activity, Trichostatin A (TSA) as a control for HDACs activity inhibition, DMSO vehicle (0.98%), and PaDef (47.3 μM). On the right side, the representative image of one of the reaction times (30 min) is shown. Different letters indicate statistically significant differences based on the vehicle (*p* ≤ 0.05) and were compared using one-way ANOVA and Tukey post hoc test (*p* ≤ 0.05). **(C)** PaDef increases Histone 3 methylation marks (dimethylation and trimethylation). On the left side is a representative western blot image. Histones were extracted from cells treated for 24 h with PaDef (47.3 μM). They were subsequently separated by SDS-PAGE and western blot assays were performed. Values were normalized to the vehicle (DMSO) for PaDef. ** (*p* ≤ 0.01) indicates the significant statistical difference based on vehicle Student’s t-test. *n* = 4. Values represent the mean ± SE of four independent experiments. Vehicle DMSO (0.98%).

It has been shown that plant AMPs can play a role as epigenetic modulators in cancer cells (Arceo-Martínez et al., 2018; Janssens et al., 2019). To evaluate whether the cytotoxic effect of PaDef was related to epigenetic modifications, we explored some epigenetic marks of Histone 3 (H3). For these assays, Jurkat cells were treated with PaDef for 24 h and then histone protein extraction was performed and the corresponding western blotting was carried out. The results showed that PaDef up-modulates (∼3 fold) the H3 global acetylation marks (H3K9, K14, K18, K23, K27) and histone 3 lysine 9 acetylation mark (H3K9me3) (Figure 7A). For these assays, sodium butyrate (NaB 3.5 mM) was used as a positive control for acetylation (Supplementary Figure S2A).

According to these results, we evaluate if PaDef affects the activity of HDACs. For this purpose, Jurkat cells were treated for 6 h with PaDef (optimal time of activity reported by Arceo-Martínez et al., 2018). The results showed that AMP significantly inhibits ∼50% HDACs activity (Figure 7B).

### The Antimicrobial Peptide PaDef increases Histone 3 Methylation Marks (Dimethylation and Trimethylation)

Further, methylation marks (H3K9me2 and H3K9me3) on H3 were measured. The results showed that PaDef up-modulates the expression of dimethylation (H3K9me2, ∼1.7 fold) and trimethylation (H3K9me3, ∼1.7 fold) marks of lysine 9 on H3 (Figure 7C). For these assays, NaB (3.5 mM) was used as a negative control for methylation (Supplementary Figure S2B).

Additionally, other epigenetic marks such as the H3 lysine 4 methylation (H3K4me3) and the H3 serine 10 phosphorylation (H3S10P) were analyzed; however, none of them were modified (data not shown).

### Cytotoxicity of Doxorubicin and the Combination With PaDef

**FIGURE 8:**
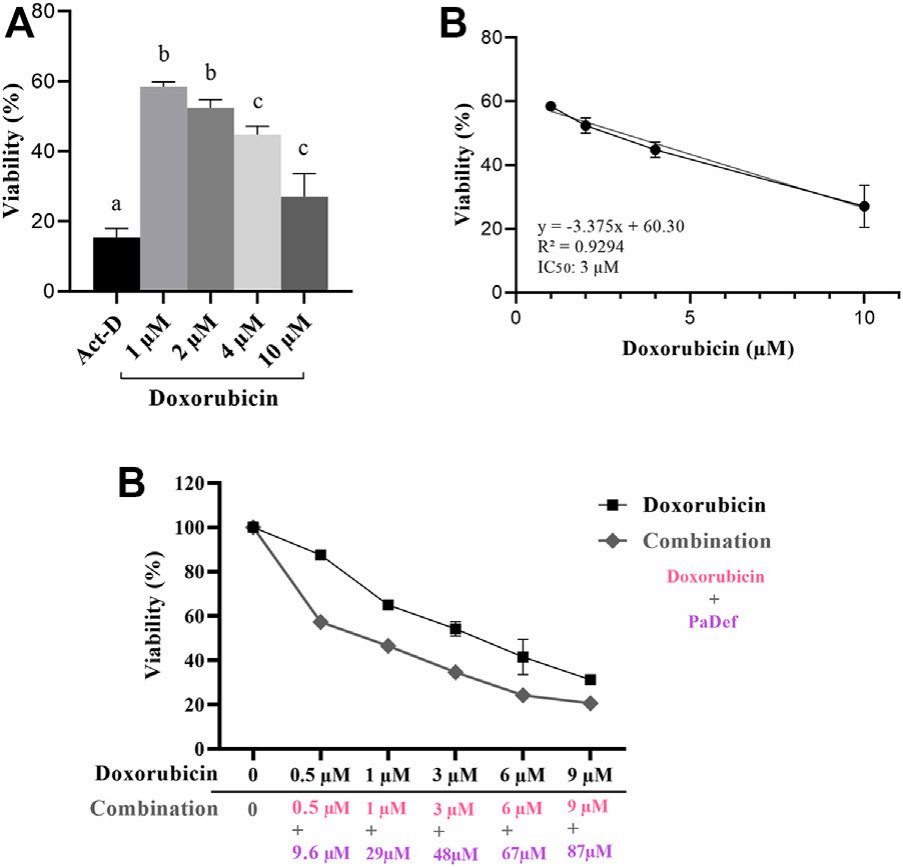
Synergistic effect of PaDef and doxorubicin in Jurkat cells. **(A)** Effect of doxorubicin on the viability of Jurkat cells. Cells were treated under increasing concentrations of the drug (1, 2, 4 and 10 μM) for 24 h and analyzed by MTT. The data show the percentage of cell viability. Actynomycin D 0.5 μM (Act-D) was used as a positive death control. Data represent the mean of three independent experiments performed in triplicate. Different letters denote significant differences within the treatments (one-way ANOVA and Tukey´s comparison, *p* ≤ 0.05). **(B)** The half-maximal inhibitory concentration (IC_50_) was calculated by linear regression analysis; IC_50_: 3 μM; R^2^ = 0.9294. **(C)** Jurkat cells were subjected to the simultaneous treatment with PaDef (9.6, 29, 48, 67 and 87 μM) and doxorubicin (0.5, 1, 3, 6 and 9 μM) and incubated for 24 h. The viable cells were determined by MTT assay and the IC_50_ was estimated. Data represent the mean of three independent experiments performed in triplicate. Different letters denote significant differences within the treatments (one-way ANOVA and Tukey´s comparison, *p* ≤ 0.05).

Finally, we evaluated the combined cytotoxic effect of PaDef with a conventional ALL chemotherapeutic, doxorubicin. The effect of doxorubicin on the viability of the Jurkat cell line was analyzed by MTT assay. Increasing concentrations of the doxorubicin (1, 2, 4 and 10 μM) were used for 24 h. From these results, the IC_50_ calculated was 3 μM (Figure 8A).

For the combined effect of PaDef and doxorubicin, concentrations were chosen considering the IC_50_ obtained from each of them. The combined treatment showed a decrease in viability ranging 40–80% (Figure 8B). Synergy analysis for the combination of treatments showed a CI (combinatorial index) of less than 1 (0.76) indicating a synergistic effect.

## Discussion

Pediatric oncology has made substantial advances in the cure of ALL. However, conventional chemotherapeutic treatments still are highly toxic and show low selectivity; also are insufficient for high-risk patients (young adults and older adults) and patients with poor prognoses (with additional mutations or epigenetic alterations); therefore, it is necessary the development of new therapies directed towards the search for molecules with specific antileukemic activity with low toxicity and high selectivity. In addition (based on the genotypic, immunophenotypic, and epigenetic characterization of the patients), these new therapies will improve the remission and survival rates of patients with leukemia. In this regard, AMPs have emerged as an interesting target for their study as therapeutic candidates against cancer, including leukemias. This interest arises from desirable characteristics of AMPs such as higher specific cytotoxicity against tumor cells and not to healthy cells, fewer adverse effects compared to conventional therapeutics, a lower tendency to develop resistance, and also, they can exhibit a broad spectrum of cytotoxic activity on tumor cells (Guzmán-Rodríguez et al., 2015; Deslouches and Di, 2017). In this work, we showed that the plant defensin PaDef (*Persea americana* var. drymifolia) is cytotoxic to the Jurkat cell line and modifies epigenetic marks, which leads to considering it as an interesting candidate in the searching of molecules against leukemias.

The AMP PaDef showed cytotoxicity on the Jurkat cells, a cell line from acute lymphoid leukemia, whose effect was concentration-dependent with an IC_50_ of 47.3 μM. In previous studies, we demonstrated that PaDef also was cytotoxic on the K562 (18.7 μM) leukemia cell line from the myeloid origin (Flores-Alvarez et al., 2018) and for the cell line MCF-7 (27.2 μM) from breast cancer (Guzmán-Rodríguez et al., 2016), being the Jurkat cell line the most resistant, and apoptosis induction the cytotoxic mechanism of the peptide on the three cell lines. Interestingly, the cytotoxic effect of PaDef on the cancer cell lines appears to be selective, since the peptide did not show a cytotoxic effect on peripheral blood mononuclear cells (Guzmán-Rodríguez et al., 2016; Flores-Alvarez et al., 2018).

Several mechanisms have been described by which AMPs from different origins can cause cancer cell death, including necrosis, autophagy, antiproliferative effects, and apoptosis (Baxter et al., 2017). In this work, we demonstrate that PaDef induces apoptosis in Jurkat cells. PaDef induced an increase in the activity of caspases 8 and 9, which activate the extrinsic and intrinsic pathways of apoptosis, respectively. Additionally, PaDef treatment induced a loss of mitochondrial membrane potential in Jurkat cells and an increase in the generation of ROS, which is characteristic of the apoptosis intrinsic pathway. Moreover, the induction of apoptosis was not dependent on changes in the cell cycle (data not shown). Dysregulation of components of the apoptotic pathway may confer a proliferative and survival advantage to cancer cells and also play an important role in the resistance to chemotherapeutic drugs and cancer recurrence (Fernald and Kurokawa, 2013; Mohammad et al., 2015). Because of this, the study of molecules capable of restoring the apoptosis process is promising in the search for new cancer therapies.

On the other hand, the study of molecules with epigenetic activity on cancer cells has gained importance, based on the fact that epigenetic alterations are involved in the development and progression of cancer. Unlike genetic mutations, epigenetic alterations are reversible, which makes them an attractive target for study (Gambacorta et al., 2019; Hajji et al., 2021). There are several leukemia treatments with an epigenetic activity that are in clinical trials (phase I to III), as well as some that have already been approved by the U.S. Food and Drug Administration for different cancer treatments. However, none of these employ antimicrobial peptides. Some of the therapies target direct DNA methylation (e.g., azacitidine and decitabine) and some others target histone modifiers, such as histone methyltransferase inhibitors (e.g., Tazemenostat and Pinomenostat), histone demethylase inhibitors (e.g., Tranylcypromine and GSK2879552), and histone deacetylase inhibitors (iHDACs, e.g., Belinostat and Vorinostat) (Kelly and Issa, 2017). iHDACs promote a decompacted state of chromatin, allowing active gene transcription to be maintained, as well as the acetylation of non-histone proteins. Several mechanisms have been proposed by which iHDACs promote their anticancer action, including cell cycle arrest, ROS production, induction of apoptosis, inhibition of DNA repair, antiangiogenic effects, or may allow acetylation of non-histone proteins (Lakshmaiah et al., 2014; Li and Seto, 2016). Of these, apoptosis induction can be activated through both apoptosis pathways (intrinsic and extrinsic). Additionally, it is known that iHDACs can activate apoptosis through the production of ROS, which has been demonstrated in several leukemia cell lines (Jurkat, ML-1, U937, HL-60, K-562) (Eckschlager et al., 2017). In concordance, we demonstrated that PaDef inhibits HDACs activity in the Jurkat cell line, whereas increases H3K9Ac. Moreover, as mentioned above, PaDef induces ROS production and triggers apoptosis through both intrinsic and extrinsic pathways. Therefore, we propose that cytotoxic PaDef activity may involve its act as an iHDAC.

Interestingly, the apoptotic activity of PaDef was not blocked by the pan caspase inhibitor Z-VAD-FMK (Figure 4), i.e., the cytotoxic activity of PaDef was maintained, regardless of caspase blockade. This behavior was already described for an iHDAC, suberoylanilide hydroxamic acid (SAHA) in the T-cell ALL cell line (CEM-CCRF) and in hepatocarcinoma cells (HepG2). SAHA induces mitochondrial membrane damage, with subsequent cytochrome c release, through ROS production and does not require activation of caspases 8 and 3. In this case, mitochondrial membrane damage was achieved by cleavage (activation) of the proapoptotic member of the Bcl-2 family named Bid (Ruefli et al., 2001; Emanuele et al., 2007). Therefore, it is necessary to analyze the pro-apoptotic members of the Bcl-2 family that may be involved in caspase-independent activation of apoptosis by PaDef in Jurkat cells.

Regarding the increase in methylation marks (H3K9me2 and H3K9me3) in the Jurkat cell line induced by PaDef treatment, there is no direct correlation between elevated H3 acetylation marks and an increase in H3 lysine 9 methylation marks. However, several studies indicate that iHDAC treatments have an impact on histone methylation status and may also induce changes in the expression of histone modulators so that the effects at the chromatin level and structure are not yet fully delineated (Hull et al., 2016). Regarding the effects of iHDAC on H3, changes have been found in marks such as H3K27me3, H3K4me3, or H3K9me3 (Huang et al., 2011; Sanders et al., 2011; Valdez et al., 2015; Ciesielski et al., 2020). For example, the treatment with the depsipeptide romidepsin, which is an iHDAC, in PEER and SUPT1 cells (T-cell ALL and T-cell lymphoblastic lymphoma, respectively) increases the acetylation status of H3 and in turn, increases the trimethylation mark of lysine 27 on H3 (H3K27me3), and this compound also induces apoptosis and favors ROS production, which is associated with mitochondrial membrane damage (Valdez et al., 2015). The effect of romidepsin agrees with the effect of PaDef here reported.

So far, epigenetic therapy including iHDACs, administered as monotherapy, has shown promising preclinical results; however, clinical trials have had limited success due to moderate response, lack of specificity, and toxic effects in patients. However, the combination of iHDACs with conventional chemotherapeutic agents has shown synergistic effects and decreased resistance to conventional treatments. The use of this type of treatment has been increasing in recent years based on the search for specific and non-cytotoxic iHDACs (Sun et al., 2019; Hontecillas-Prieto et al., 2020). In this regard, as a first approach to the use of PaDef as an iHDACs we tested the combined effect of PaDef with one of the conventional chemotherapeutics for leukemias: Doxorubicin. The resulting synergistic effect between the two treatments indicates that PaDef could be a promising peptide in combined therapy against leukemias, based on its epigenetic effect on the Jurkat cell line as an iHDAC.

A complete understanding of molecules with iHDAC activity is still far off and requires to be studied at multiple levels because their effects are reflected not only at the chromatin level (histone acetylation and histone methylation changes), but may also involve acetylation changes in non-histone proteins, which in turn can regulate cell signaling pathways, culminating not only in changes in gene expression. The probable interaction of PaDef with intracellular target is very interesting; however, it has been reported that the AMPs cecropin A, magainin 2, and indolicidin can be degraded by 100% after 24 h using cytosolic extracts of human erythrocytes (Starr and Wimley, 2017). Until now, we do not know if PaDef could be also degraded by intracellular proteases.

## Conclusion

Results from this work strongly suggest that PaDef can be an attractive cytotoxic plant antimicrobial peptide against ALL, whose antiproliferative activity could be related to epigenetic modulation, which can lead to the chromatin compaction-decompaction promoting gene expression or repression. However, further studies are necessary to correlate epigenetic marks with the transcription of specific genes. These novel effects have not been described until now for plant defensins.

## Data Availability

The original contributions presented in the study are included in the article/Supplementary Material, further inquiries can be directed to the corresponding author.

## Abbreviations

Act-D, Actinomycin D; ALL, Acute Lymphoblastic Leukemia; AMP, Antimicrobial Peptide; CI, Combination index; DMSO, Dimetil sulfoxide; H3, Histone 3; H3K9Ac, Acetylation of lysine 9 on Histone 3; H3K9me2, Dimethylation of lysine 9 on Histone 3; H3K9me3, Trimethylation of lysine 9 on Histone 3; HDAC, Histone deacetylase; IARC, International Agency for Research on Cancer; IC_50_, Inhibitory concentration 50; iHDAC, Histone deacetylase inhibitor; PaDef, Persea americana var. Drymifolia; PBS, Phosphate buffered saline; PMA, 4alpha-Phorbol 12-myristate 13-acetate; ROS, Reactive Oxygen Species; WHO, World Health Organization.
