# Supplementary figures and images for: PaDef (Persea americana var. drymifolia), a Plant Antimicrobial Peptide, Triggers Apoptosis, and Induces Global Epigenetic Modifications on Histone 3 in an Acute Lymphoid Leukemia Cell Line
Source: Front Mol Biosci. 2022 Jan 24;9:801816. doi: 10.3389/fmolb.2022.801816 (PMC8820506; doi:10.3389/fmolb.2022.801816)

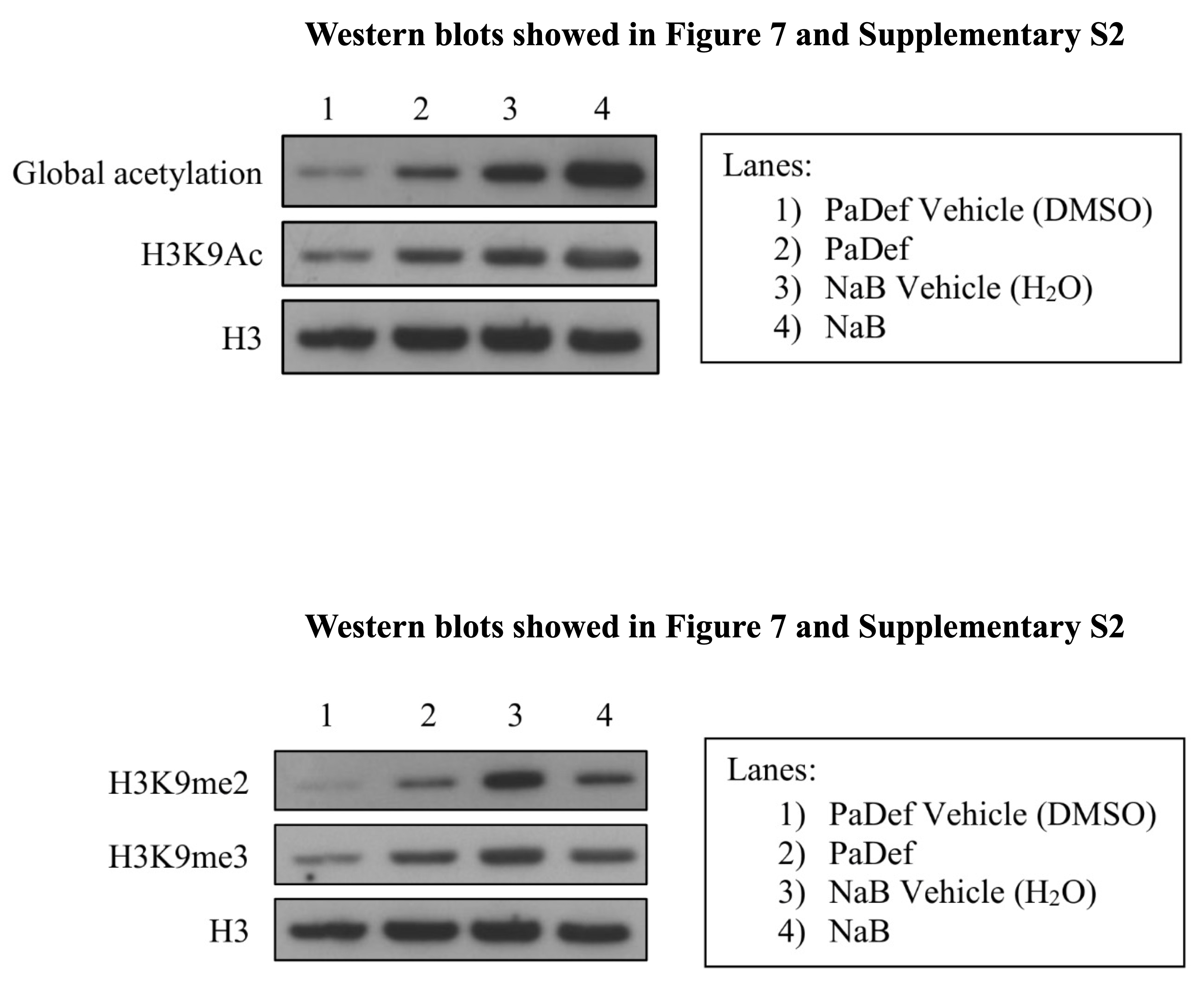

Supplement: Supplementary file 1 [file Image3.TIFF]

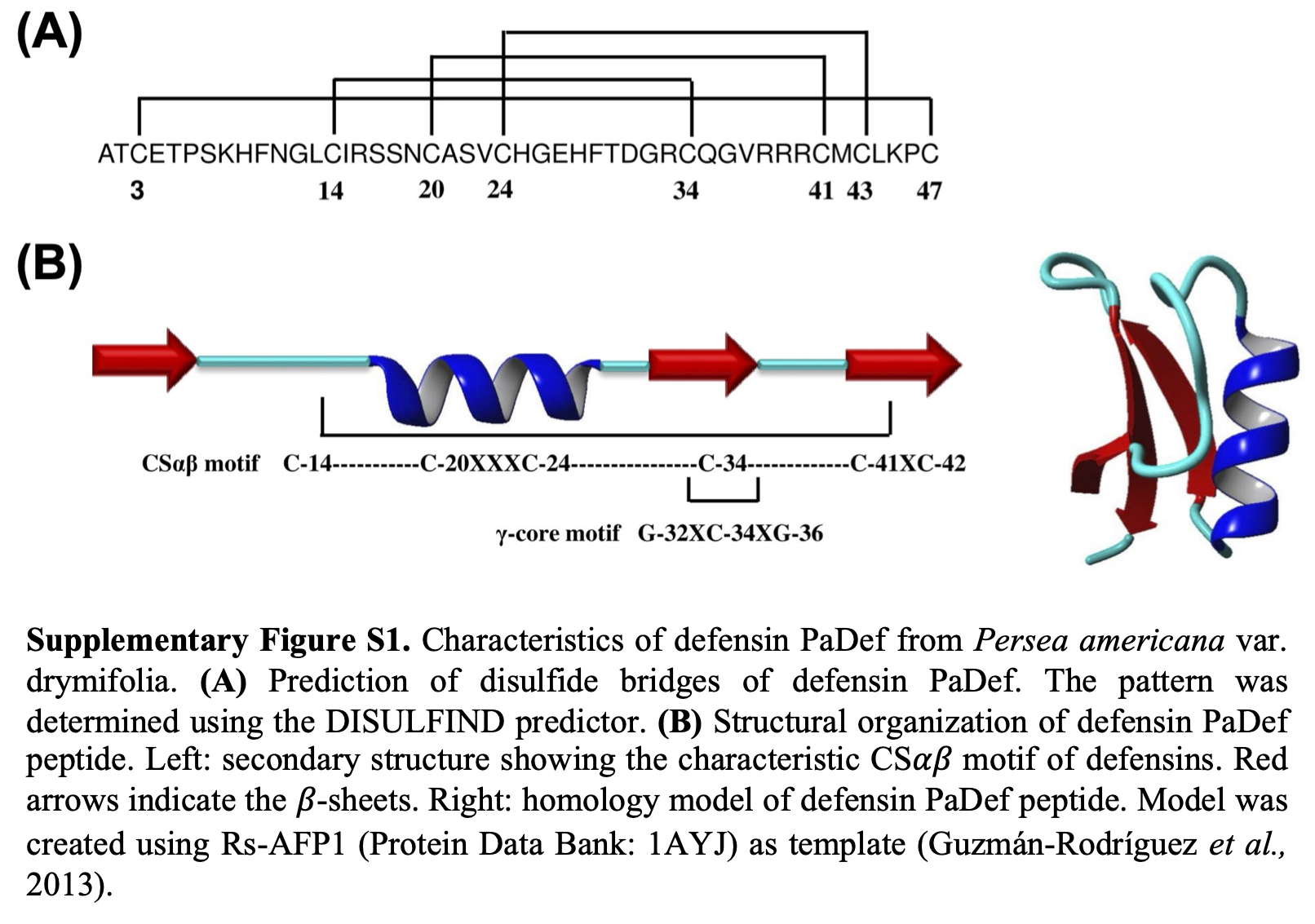

Supplement: Supplementary file 2 [file Image1.tiff]

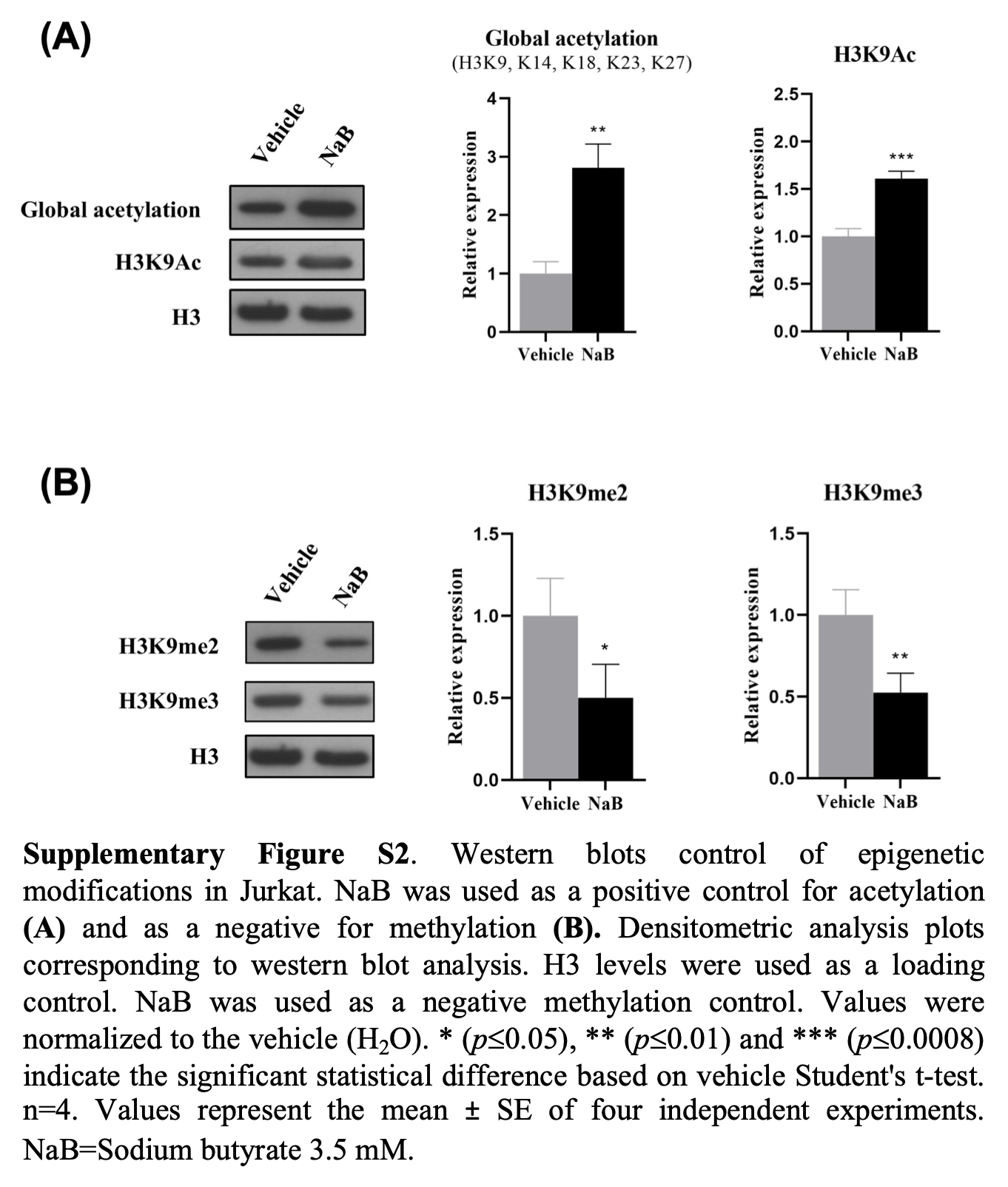

Supplement: Supplementary file 3 [file Image2.tiff]
